# The Kleine-Levin Syndrome: A Rare Disease with Often Delayed Diagnosis—A Report of Two Cases in the Department of Neurology of the University Hospital of Cocody (Côte d'Ivoire)

**DOI:** 10.1155/2016/8929413

**Published:** 2016-02-17

**Authors:** Berthe Assi, Constance Yapo-Ehounoud, Mohamed Ben Allaoui Baby, Evelyne Aka-Diarra, Muriel Amon-Tanoh, Christian Tanoh

**Affiliations:** Department of Neurology, University Hospital of Cocody, Abidjan, Côte d'Ivoire

## Abstract

The Kleine-Levin syndrome is a rare pathology characterized by recurrent episodes of hypersomnia associated with behavioral and cognitive disorders with, among others, hyperphagia and hypersexuality. The disease mainly affects young males. A few studies mention cases that occurred in Africa, especially in Côte d'Ivoire. In this paper, we report the very first two cases observed in the Neurology Department of the University Hospital of Cocody. The diagnosis was clinical, based on the recurrence of hypersomnia, cognitive and behavioral disorders during the periods of hypersomnia, and the return of patients to normal state between episodes. This diagnosis was delayed due to failure to understand the pathology, thereby leading patients to wandering. In fact, the two patients were consulted, respectively, 3 years and 6 years after the hypersomnia began. The objective was to report the very first cases observed in the Neurology Department of the University Hospital of Cocody, Côte d'Ivoire.

## 1. Introduction

The Kleine-Levin syndrome (KLS) is classified among rare diseases [1, 2]. It belongs to the central hypersomnias said to be recurrent and defined according to the International Classification of Sleepiness Disorders, 3rd version (ICSD-3), revised in 2014. For the ICSD-3, the Kleine-Levin syndrome is a disorder characterized by recurrent episodes of hypersomnia and hyperphagia (rapid consumption of a large amount of food), usually with onset in early adolescence in males but occasionally in later life and in women. A monosymptomatic form of the disorder with hypersomnia only can occur without binge eating or hypersexuality [1]. Usually affecting adolescent males, episodes normally last up to a few weeks and terminate with total and spontaneous recovery. Possibly first reported by Brierre de Bosmont in 1862, the condition however received its name from Willi Kleine who, in 1925, reported a series of cases of periodic hypersomnia and also Max Levin who described a case of periodic hypersomnia and excessive appetite in 1930 [2]. The exact prevalence remains unknown. But it seems very rare: around one to two cases per million. All cases are not published. A review of the international literature of language between 1962 and 2004, by Arnulf et al. [2], estimated the number of published cases at 186. According to these authors, the annual incidence was 2.7 in 1970, 3.5 in 1980, and 5.8 in the 1990s. For these authors, this growth would rather correspond to the extension of the global scientific communication than an actual increase in the prevalence of the syndrome. Patients were described around the world, including Asia and Africa, with curiously one-sixth of the world's cases described in Israel [1, 2]. Its etiology remains unknown. The genetic trail was discussed in the presence of familial cases [1, 2]. The disease mainly affects young males [1, 2]. Its diagnosis is clinical [1, 3, 4].

Only one case of Kleine-Levin in a young girl of 14 was reported in Nigeria by Malomo et al. in 1998 [4]. No case in Côte d'Ivoire was reported so far. In reporting the very first two cases of Kleine-Levin syndrome diagnosed in our service, we aim at bringing our contribution to science.

## 2. Case 1

A 25-year-old Ivorian young man, with no job, was received for consultation in the Neurology Department on 9 January, 2012, for hypersomnia associated with behavioral disorders and chronic headaches. In his background, neither prenatal pathologies nor psychomechanic development disorders were noted. The patient had no antecedent with toxic mania or psychopathology. His hypersomnia was said to have begun in the evening of 27 June, 2008, four years earlier, at the age of 18 after he had taken the national baccalaureate examination. The hypersomnia was triggered by the consumption of alcoholic beverage (a 66cl-bottle of beer) and influenza-like syndromes. It lasted an average of 18 hours. It was accompanied with behavioral disorders with kinds of aggressiveness associated with an eccentric attitude, visual hallucinations related to death, forced thoughts of death, memory loss, and chronic headaches. These episodes lasted about three weeks, and then the patient quickly found his usual lucidity. During these episodes of hypersomnia, the patient had an unusual bulimia. The patient had no known history of substance abuse or prior psychiatric disorder. The neurological examination was normal. The Cranioencephalic computed tomography (CT) was normal. A polysomnography could not be performed due to shortage of financial means. The diagnosis of the Kleine-Levin syndrome was selected. The patient was placed under modafinil in a dosage of 100 mg a day through oral route. A month later, he reported a reduction in the duration of hypersomnia, but with the persistence of visual hallucinations, forced thoughts of death. Since the beginning of 2015, there has been a resurgence of hypersomnia episodes going from once a year to three episodes in the same year. Because of the high cost of the modafinil (200 dollars), the treatment was carried out occasionally.

## 3. Case 2

A young Ivorian man of 21 years old in eleventh grade was received in consultation on 4 February, 2015, for chronic headaches and sleepiness. The troubles started in 2009, six years before when the patient was 15 years old. In his background, neither prenatal pathologies nor psychomechanic development disorders were noted. However, we noted chickenpox in his childhood. The patient was not an alcohol addict. The crisis occurred after the first term of school exams. The hypersomnia occurred 3 to 4 times a year. The patient slept at least for 24 hours during 2 to 10 days, and then he found his usual lucidity. During the hypersomnia, his sleepiness was disturbed by nightmares in which he said he was at the cemetery or funeral wakes. He also had visual hallucinations associated with death. He had cognitive disorders, difficulties in concentration during reading, and difficulties in completing two tasks at a time (said “confused”). The patient had a sense of unreality, with sensation of disconnection between the body and the spirit, and the feeling of being in a dream. Behavioral disorders were noted such as bulimia, hypersexuality with increase in the libido, and sex-oriented thoughts. The episodes lasted 9 to 10 days and the patient recovered his usual lucidity. Between episodes, periods of respite were 5 to 6 months, during the first 4 years, and became shorter, or an average of four months for 2 years. He exhibited scarce time-laps of insomnia in between attacks.

The physical examination was normal. The electroencephalogram (EEG) has objectified potential acute in bifrontal puffs. The Cranioencephalic CT scan in the search of a brain injury was normal. A polysomnography could not be performed. The diagnosis of the Kleine-Levin syndrome was selected. The patient received treatment such as valproic acid, at the dose of 1 g through oral route.

## 4. Discussion

### 4.1. Epidemiological and Demographic Data

The Kleine-Levin syndrome is often said to be a rare disease with a prevalence of 1 to 2 cases per million of inhabitants. We report the very first cases observed in our service. This syndrome is a typical example of a little known disease, little taught and little diagnosed and with often delayed diagnosis. It leaves the patient, his family, and their practitioner often isolated and disabled. It leads to the increase in the diagnostic procedures, examinations (sometimes invasive), and clinical trials [1]. As described in the literature, cases found in series are also known as a wandering that explains the delay in the diagnosis. Men would be 2 to 3 times more often affected than women [1, 2]. Our patients were both males. The disease starts in the majority of patients (81%) in the teens, with a median of 15 years, and in some before puberty or in adulthood, according to Arnulf et al. [2]. The age of onset was 19 years and 15 years, respectively, for our first and second patient, which is consistent with the literature [1, 2]. For Arnulf et al., most of the cases are sporadic, as it is in our patients. But familial cases have been described [1, 2].

### 4.2. Clinical Data

The diagnosis is by far clinical according to Arnulf et al. [1, 2].

As described in the literature, our patients had recurrent hypersomnia, with sudden onset [2, 5]. This hypersomnia was long, 24 hours a day. This recurrent hypersomnia is associated with a feeling of unreality, apathy, cognitive disorders, and sometimes confusion. In half the cases, it may be associated with unusual gluttony, sexual or social disinhibition, anxiety disorders, compulsion or mood, and rarely hallucinations [1, 2, 4, 5]. Our two patients also presented the clinical features usually described: hypersomnia, bulimia, and behavioral and cognitive disorders.

Suggestive signs of meningeal irritation (without neck stiffness) are observed in half of the cases, with type of headaches, photophobia, fever, and occasionally nausea [2]. Between these meningeal signs, only headache was found in our patients.

The duration of an episode varies, according to Arnulf et al. [2], from 2 to 270 days, with a clear median, whatever the studies, around 10 days. For Rezvanian and Watson [5] and Ramdurg [4], the episode duration varies from 1 week to 1 or 2 months. In our series, the episode lasted 3 weeks for the first case and 2 to 10 days for the second case.

In between episodes, patients recover for several months their normal state [4, 5]. This period of respite has an average from 3.5 to 4 months and would be very variable from one individual to another. According to Arnulf et al. this period of respite, which was initially 6 months for our second patient, during the first four years, rose to four months during the last two years.

The gradual or rapid return to normality at the end of episode is characteristic of the Kleine-Levin syndrome. Our patients effectively recovered a clinical normal state between episodes, as reported in the literature by several authors [1, 2, 4–6]. As described in literature [2], the second patient sometimes presented insomnia out of the hypersomenia episode.

The disease progresses in spurts, interspersed with periods where the patient is normal, for a median duration of 13.5 years, according to Arnulf et al. [2]. For these authors, the duration of the disease is longer in male patients with hypersexuality and in the patients whose disease began after puberty. In our series, the first case began his disease after puberty, and the second case had a sex-oriented thought. In addition, in this second case the hypersomnia episodes became more frequent. We could think that these 2 patients run the risk of having their disease persistent for long enough, but there is no evidence to back such an assertion up [2].

### 4.3. Diagnostic Tests

The cerebral morphological imaging (CT and MRI) is generally normal [2, 4]. This is the case in our study. A functional imaging study of brain scan during the symptomatic period objectified frequent transient hypoactivity in the thalamic region and to a lesser degree of the hypothalamus and the frontal and temporal regions [6]. These cerebral hypoperfusion areas support the hypothesis of diencephalic and clinical symptoms of the Kleine-Levin syndrome.

The EEG is the examination which is the most frequently abnormal in the KLS. It shows during the episode, in 70% of cases, either a slowdown in the basic alpha rhythm (compared to their rhythm outside of the episode) or slow wave delta or theta, isolated or in flashes, generally temporal or temporofrontal [2]. For Ramdurg, there are often discrepancies, sometimes with points that can wrongly be evoked in an epileptic [4]. This is the case of our second patient. In our series, especially for case 2, the Kleine-Levin syndrome was associated with electrical abnormalities in acute type bifrontal puffs.

The polysomnographic recordings, made during the access [7], showed an extension of the total duration of sleepiness, however with flow and normal sleepiness pattern. Sometimes we could notice a reduction in the latency of paradoxical sleepiness [5, 7].

### 4.4. Factors Triggering Episodes

The episodes were triggered in the first patient by influenza and ingestion of alcoholic beverage. In the second patient, episodes occurred at the end of term examinations, hence triggered by stress.

### 4.5. Etiology and Vulnerabilities

No definitive cause has been established [2, 4].

There are different vulnerabilities to the syndrome: being male (2/3 of patients), prenatal and developmental problems (RR: 6.5), being Ashkenazi (HR 6), and genetic factors (5% of multiplex families). Rezvanian and Watson report that the association with a particular HLA genotype has not been confirmed on large series. The only factor identified in our series remains male gender. The genetic testing was not performed due to lack of technical facilities. Because of the rarity of the disease (the diagnosis remains essentially clinical) diagnostic errors are common and therefore leading to wrong reference to the multiplicity of diagnoses. According Huang et al. there are many patients with episodic alteration of sleepiness, appetite, and behavior with a response to similar treatment to the classic Kleine-Levin syndrome, which, however, do not fit the classical description for the diagnosis of this disease [8].

### 4.6. Treatment

Various symptomatic and prophylactic treatments offered remained disappointing [2]. Indeed, stimulants symptomatic treatments of awakening, such as amantadine, modafinil, and amphetamines during the access, have low efficiency rate. No single drug therapy has been successful, in spite of the various psychotropic agents, including lithium, anticonvulsants, and antidepressants [9]. The modafinil showed a profit with the effect of shortening the duration of symptomatic periods but does not affect the recidivism rate [9]. Our first case received modafinil. According to him, the duration of the following hypersomnia was shorter; however hallucinations persisted. It easily matches the finding of Yao et al. However the works by Das et al. demonstrated the effectiveness of lithium. Other authors like Rezvanian and Watson showed the effectiveness of other molecules especially clarithromycin in the treatment of Kleine-Levin syndrome. Indeed, their study showed a short-term beneficial effect of clarithromycin. The limited nature in time of effect limits the practical usefulness of this drug even though it involves the part of the GABA-A receptor in the pathophysiology of Kleine-Levin syndrome. In all cases it is necessary, according to Oliveira et al., as well as other authors, to perform double-blind clinical trials and pharmacological treatment for Kleine-Levin syndrome [6, 10–12].

Due to the low effectiveness of amantadine during episodes (other stimulants are not very useful), as well as mood stabilizers (lithium, valproate, but not carbamazepine) in background processing, for some authors like Rezvanian and Watson, no treatment is often preferable.

## 5. Conclusion

The Kleine-Levin syndrome is a rare neurological pathology. It most often affects young male adults. It is insufficiently described in Africa. Its pathogenesis remains unsolved. The diagnosis of the Kleine-Levin syndrome is essentially clinical yet often delayed. It is based on a recurrent hypersomnia, associated with an unusual overeating, cognitive, and behavioral disorders, symptoms presented by our two patients.

The treatment is often disappointing. As a result, no treatment is often recommended. Some treatment such as modafinil proved effectiveness in the hypersomnia whereas lithium or sodium valproate showed a mood stabilizer effect.
